# The binding orientations of structurally-related ligands can differ; A cautionary note

**DOI:** 10.1016/j.neuropharm.2017.01.023

**Published:** 2017-06

**Authors:** Marc-David Ruepp, Hao Wei, Michele Leuenberger, Martin Lochner, Andrew J. Thompson

**Affiliations:** a Department of Chemistry and Biochemistry, University of Bern, Bern, Switzerland; b Department of Pharmacology, University of Cambridge, Cambridge, UK; c Institute of Biochemistry and Molecular Medicine, University of Bern, Bern, Switzerland

**Keywords:** Cys-loop, Ion channel, Receptor, Radioligand, 5-HT_3_, Agonist, Antagonist, Crystal, Binding, Granisetron, Tropisetron, Ligand, Structure, Synthesis, 5-HT, 5-hydroxytryptamine, nACh, nicotinic acetylcholine, GABA, gamma-aminobutyric acid, HEK, human embryonic kidney, AChBP, acetylcholine binding protein, 5HTBP, an AChBP mutant modified to resemble the 5-HT_3_R binding site, SAR, structure-activity relationship

## Abstract

Crystal structures can identify ligand-receptor interactions and assist the development of novel therapeutics, but experimental challenges sometimes necessitate the use of homologous proteins. Tropisetron is an orthosteric ligand at both 5-HT_3_ and α7 nACh receptors and its binding orientation has been determined in the structural homologue AChBP (pdbid: 2WNC). Co-crystallisation with a structurally-related ligand, granisetron, reveals an almost identical orientation (pdbid; 2YME). However, there is a >1000-fold difference in the affinity of tropisetron at 5-HT_3_ versus α7 nACh receptors, and α7 nACh receptors do not bind granisetron. These striking pharmacological differences prompt questions about which receptor the crystal structures most closely represent and whether the ligand orientations are correct. Here we probe the binding orientation of tropisetron and granisetron at 5-HT_3_ receptors by *in silico* modelling and docking, radioligand binding on cysteine-substituted 5-HT_3_ receptor mutants transiently expressed in HEK 293 cells, and synthetic modification of the ligands. For 15 of the 23 cysteine substitutions, the effects on tropisetron and granisetron were different. Structure-activity relationships on synthesised derivatives of both ligands were also consistent with different orientations, revealing that contrary to the crystallographic evidence from AChBP, the two ligands adopt different orientations in the 5-HT_3_ receptor binding site. Our results show that even quite structurally similar molecules can adopt different orientations in the same binding site, and that caution may be needed when using homologous proteins to predict ligand binding.

## Highlights

• The drugs granisetron and tropisetron are structurally similar.
• Crystals of them bound to AChBP suggest they have similar binding orientations.
• At 5-HT_3_R, the effects of mutagenesis indicate that their orientations differ.
• SAR on both of these drugs also supports different orientations.

## 1 Introduction

Tropisetron (e.g. Navoban^®^, ICS 205-930), granisetron (e.g. Kytril^®^, Sancuso^®^, Granisol™) and other structurally-related drugs are used to alleviate the symptoms of nausea and vomiting following general anaesthesia, cancer chemotherapy and radiotherapy (Thompson, 2013). The therapeutic effect of these is due to their high-affinity competitive block of 5-HT_3_ receptors in the gut and brain stem.

**Fig. 1:**
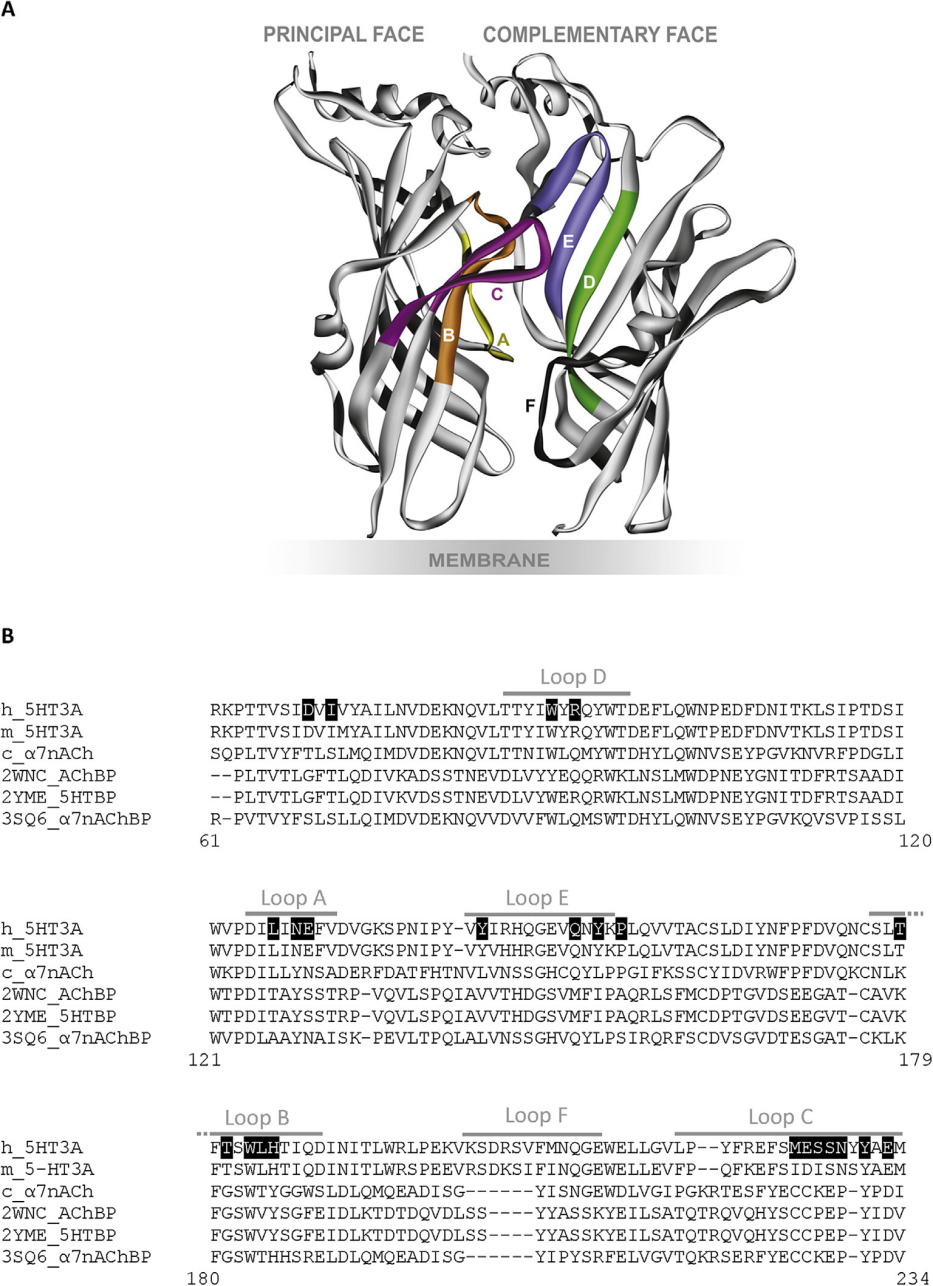
Positions of the residues mutated in this study. (**A**) A cartoon showing the orthosteric binding site of the 5-HT_3_ receptor formed by binding loops A–F. The extracellular binding site is found at the interface of two adjacent subunits. For clarity only two subunits are shown. (**B**) An amino acid sequence alignment showing the positions of mutated residues (white text, black boxes). The six recognised binding loops are shown as grey lines. The proteins are sequences from human 5-HT3A (P46098), mouse 5-HT3A (Q6J1J7), chick α7 nACh (F1P4Y5) and sequences taken from the AChBP crystal structures 2YME, 2WNC and 3SQ6. c_α7nACh and h_5HT3 are the sequences of receptors used in the binding studies presented here. EMBOSS Needle shows that the sequence of 2WNC_AChBP has a closer identity and similarity to c_α7nACh (Id = 27.4%; Sim = 41.8%) than to h_5HT3 (Id = 19.4%; Sim = 31.8%) (Rice et al., 2000). To facilitate comparisons with previous work, the numbering used throughout this manuscript refers to residues at equivalent positions of the mouse 5-HT3A subunit (Q6J1J7).

5-HT_3_ receptors belong to the Cys-loop family of transmembrane ligand-gated ion-channels that are responsible for fast synaptic neurotransmission in the central and peripheral nervous systems. All members of this family are composed of five subunits, each of which contains an extracellular, a transmembrane and an intracellular domain (Miller and Smart, 2012, Thompson et al., 2010). Binding of tropisetron and granisetron to extracellular binding sites blocks the action of the native agonist 5-HT. These binding sites are at the interface of two adjacent subunits and form a hydrophobic cavity that is composed of amino acids from loops A - C in the principal subunit interface and loops D - F in the complementary subunit interface (Fig. 1).

Crystallisation studies have attempted to evaluate the binding orientations of several 5-HT_3_ receptor ligands, but all of these have been performed on a close structural homologue rather than the native receptor. For example, cocaine (pdbid: 2PGZ) and tropisetron (2WNC) were crystallised in the binding site of acetylcholine binding protein (AChBP), while granisetron (2YME) was crystallised with an AChBP mutant containing two loop D amino acids substitutions that increased its affinity for this ligand (Hansen and Taylor, 2007, Hibbs et al., 2009, Kesters et al., 2013). Other studies have sought to explore the binding orientation of granisetron using homology modelling and ligand docking, but the majority of these are over a decade old and do not benefit from our improved understanding of the receptor family or developments in the computer software used to model them (reviewed in Thompson et al., 2010). For example, the apo crystal structure of the mouse 5-HT_3_ receptor was published in 2014 and has provided an unprecedented insight into the native structure in the absence of a ligand (Hassaine et al., 2014).

In addition to high affinity competitive antagonism at 5-HT_3_ receptors, tropisetron is also reported to bind to the α7 nACh receptor which belongs to the same Cys-loop receptor family (Macor et al., 2001, Papke et al., 2005). However, at α7 nACh receptors tropisetron causes activation. In contrast, at the same receptor, binding of the very close granisetron congener and 5-HT_3_ receptor antagonist LY-278,584 cannot be detected (Macor et al., 2001). Despite these pharmacological differences, in co-crystal structures with the homologue AChBP, tropisetron and granisetron adopt almost identical positions and orientations in the binding site. Given the quite distinct pharmacological properties of these ligands it is therefore unclear whether the structures are true representations of the orientations found at 5-HT_3_ or α7 nACh receptors, and if they are, which of the two receptors the AChBP co-crystal structures most closely resemble.

Here we probe the binding orientations of tropisetron and granisetron at 5-HT_3_ receptors, using a combination of bioinformatics, cysteine mutagenesis and the synthesis of ligand analogues. The results show that their orientations differ, and question whether it is appropriate to use these receptor homologues for predicting ligand binding at different members of the Cys-loop family (Thompson et al., 2017).

## 2 Results

### 2.1 Docking

**Fig. 2:**
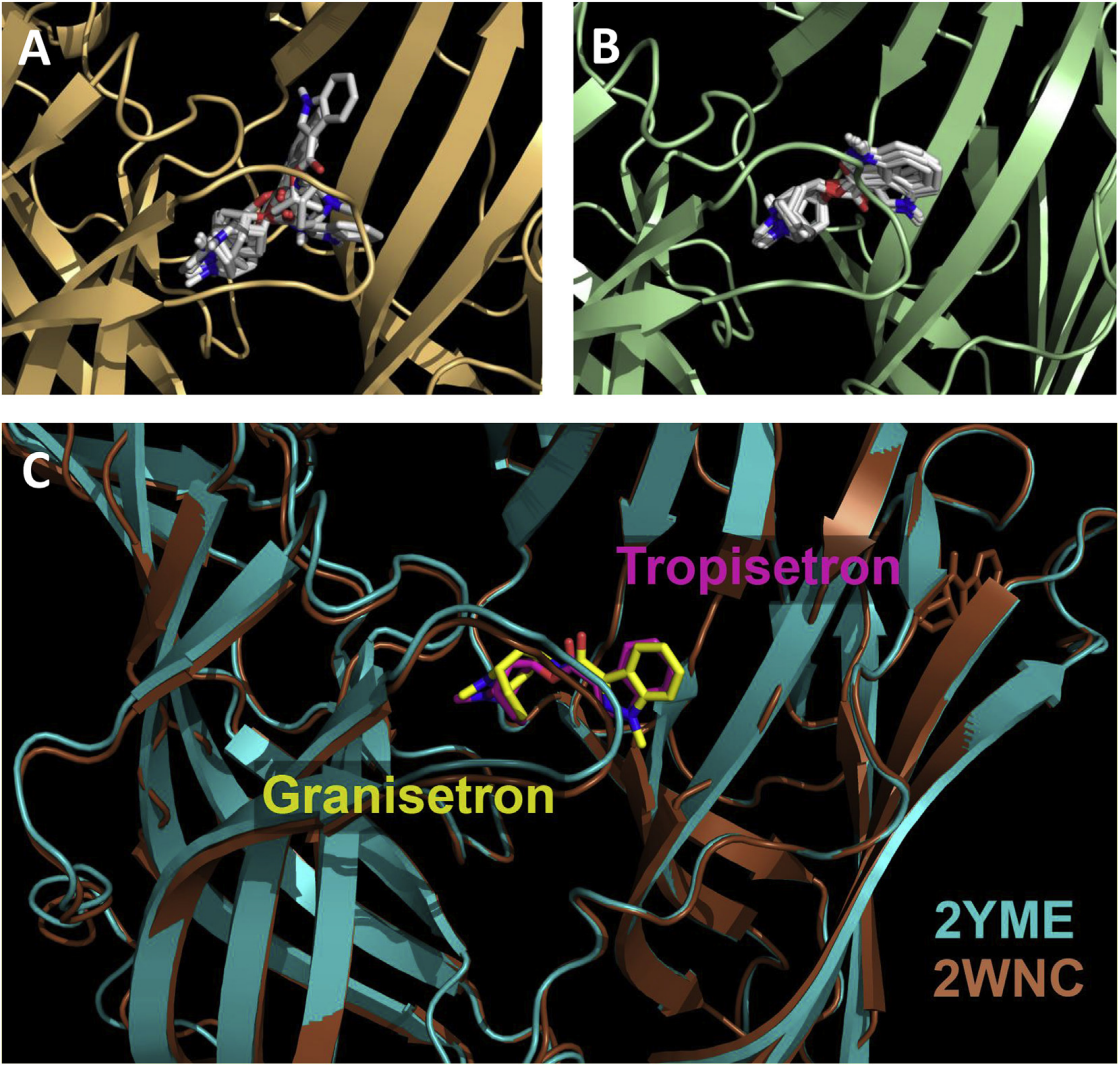
Binding of tropisetron. Predicted binding clusters for tropisetron docked into (**A**) a homology model of the 5-HT_3_ receptor binding site based upon the template 2WNC (*Model-1*), and (**B**) the crystal structure 2YME (*Model-2*). (**C**) Co-crystal structures 2WNC and 2YME show AChBP bound with tropisetron and granisetron respectively. In panels 2A & 2B all 10 predicted ligand poses are overlaid. 5-HT_3_ receptor residues within 5 Å of tropisetron in each of these poses are shown in Table 1.

To gain insights into potential binding-site interactions, residues located within 5 Å of tropisetron or granisetron were identified from four sources; (1) tropisetron in the AChBP co-crystal structure 2WNC, (2) granisetron in the AChBP co-crystal structure 2YME, (3) *in silico* docking of tropisetron into a 5-HT_3_ receptor homology model based upon 2WNC, and (4) *in silico* docking of tropisetron into 2YME (Fig. 2). From the analysis of the resultant 22 ligand orientations, a total of 33 residue positions were identified as being within 5 Å of a ligand, and were located throughout the binding loops A - E (Table 1, Fig. 1). Five of the residues were common to all predicted ligand orientations (W90, N128, W183, Y234, Y153). Sixteen other residues (K112, I127, E129, Y141, V142, Y143, V150, N152, K154, P155, T179, F180, T181, H185, A235, E236) were unique to only one of the four predictions (Table 1). The remaining 12 residues (D69, I71, R92, Q151, L126, S182, L184, M228, E229, S230, S231, N232) were distributed in at least two of the predictions. Of the 33 identified residues, 23 were mutated to cysteine.

**Table 1:**
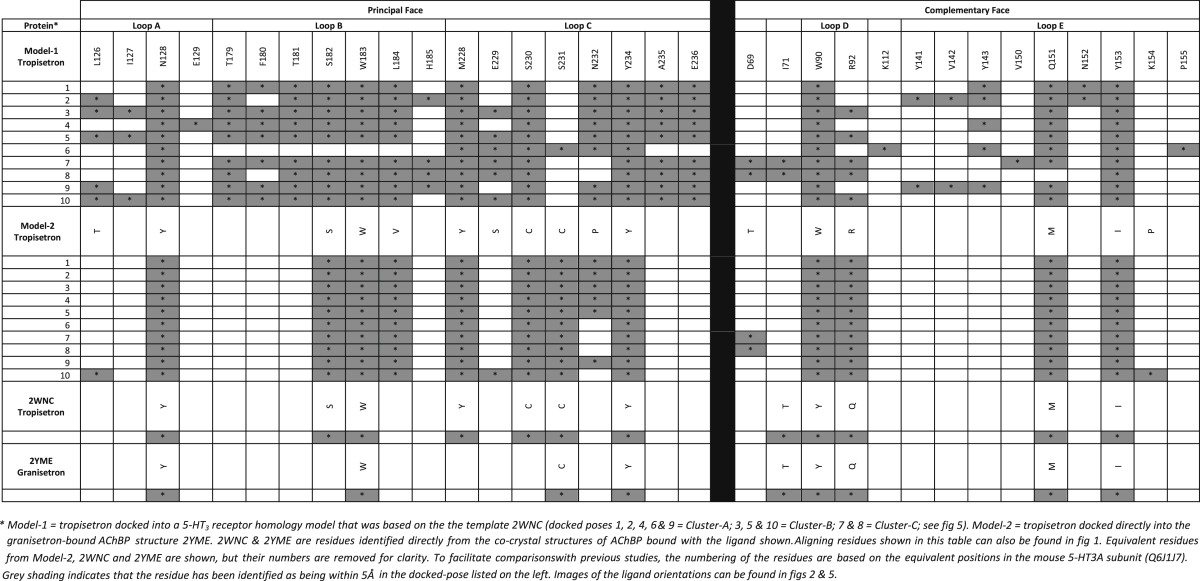
Residues predicted to be within 5 Å of tropisetron or granisetron in the 5-HT_3_A receptor binding site.

### 2.2 Radioligand binding

Untransfected HEK293 cells showed no saturable binding with [^3^H]tropisetron or [^3^H]granisetron, but upon transient expression of wild type 5-HT_3_ receptors both ligands displayed high-affinity saturable binding (Table 2, Table 3). Non-specific binding was 30.3 ± 1.8% (*n* = 8) of total for tropisetron and 6.7 ± 0.4% (*n* = 6) for granisetron. *K*_d_ values were similar to those reported elsewhere (Neijt et al., 1988, Thompson et al., 2005).

**Table 2:**
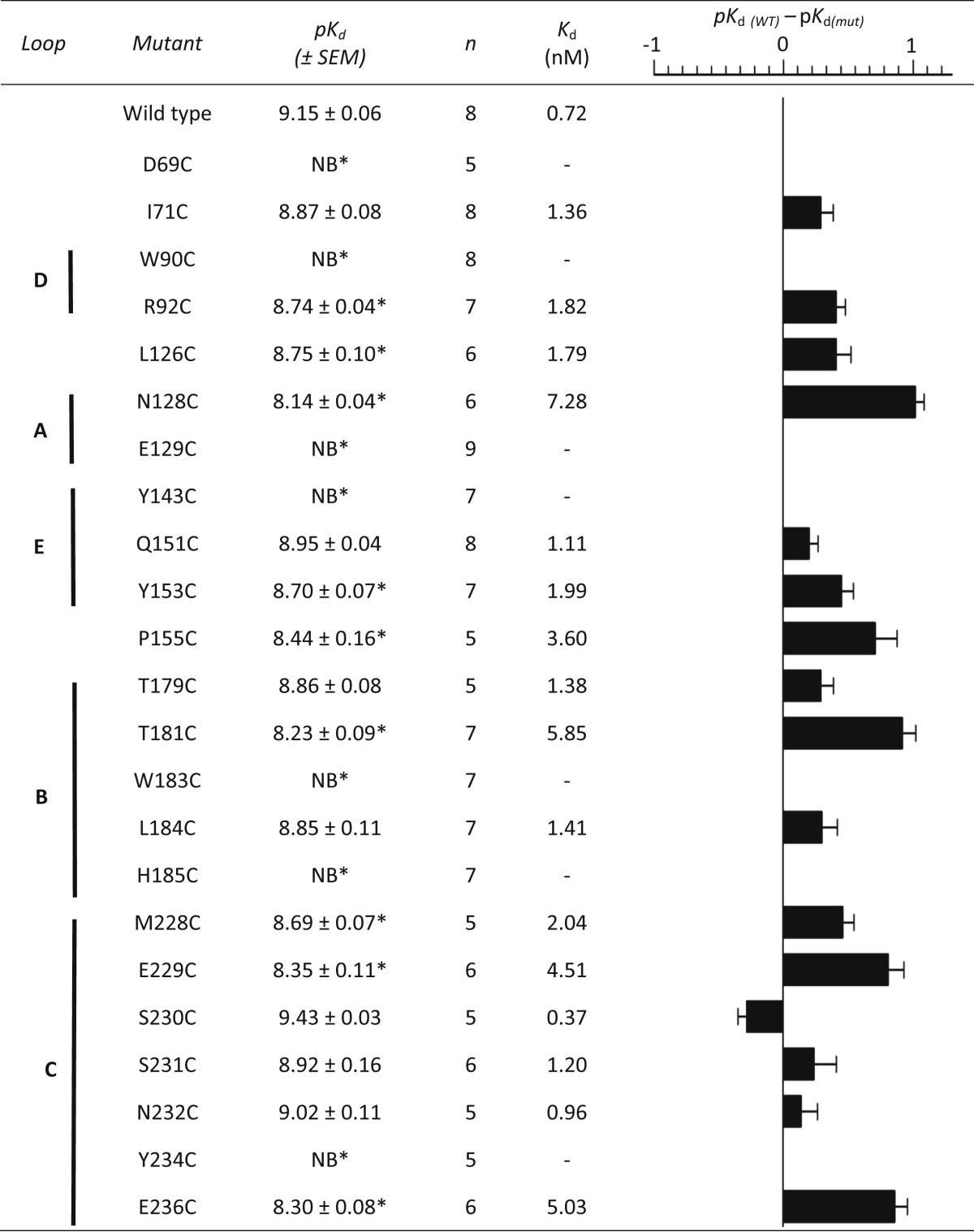
Saturation binding of [^3^H]tropisetron at 5-HT_3_A receptor cysteine mutants.

∗ significantly different (1-way ANOVA and a Dunnett's Post-Test) to wild type receptors. SED = standard error of the difference.

**Table 3:**
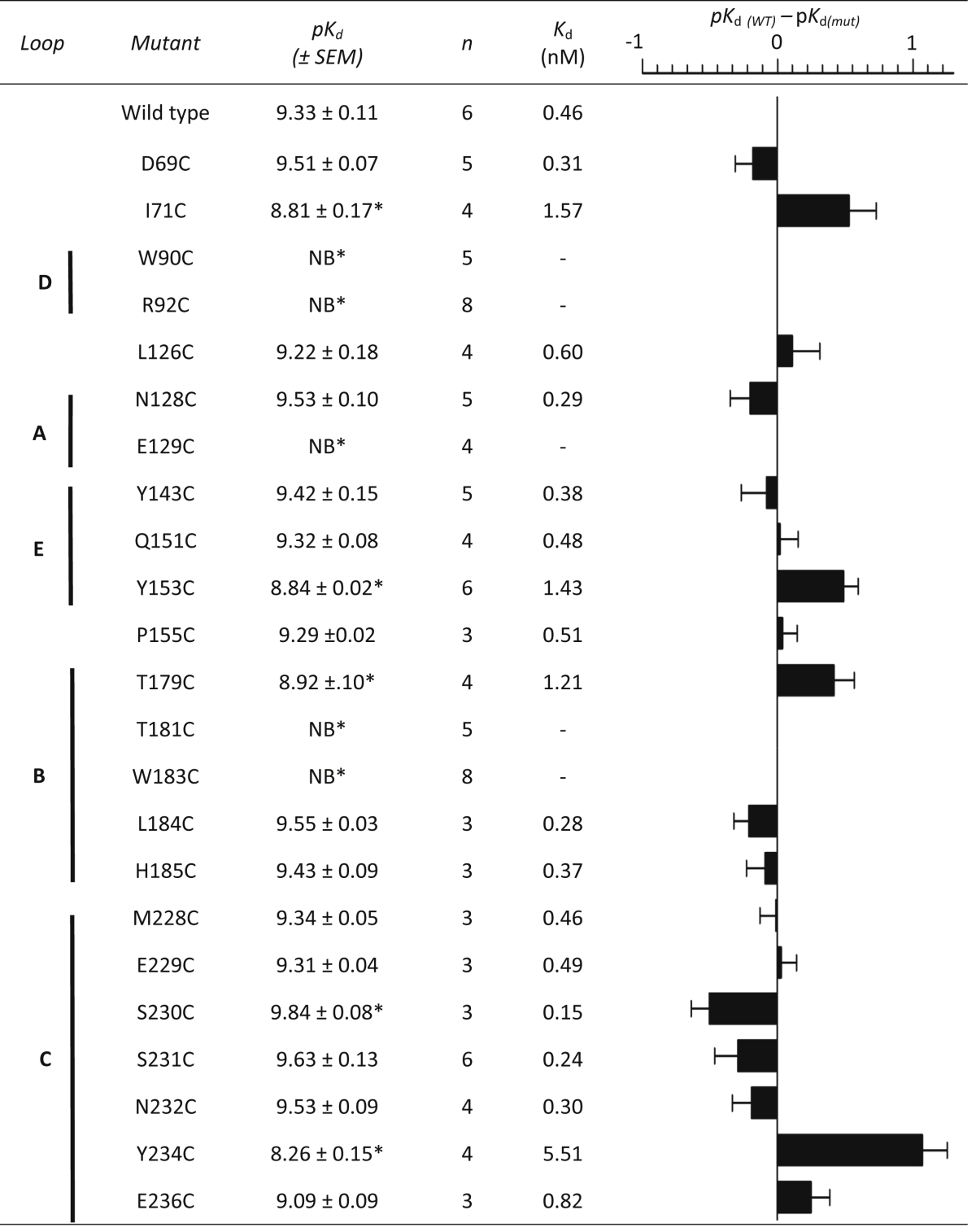
Saturation binding of [^3^H]granisetron at 5-HT_3_A receptor cysteine mutants.

∗ significantly different (1-way ANOVA and a Dunnett's Post-Test) to wild type receptors. SED = standard error of the difference.

### 2.3 Effects of mutations on tropisetron binding

**Fig. 3:**
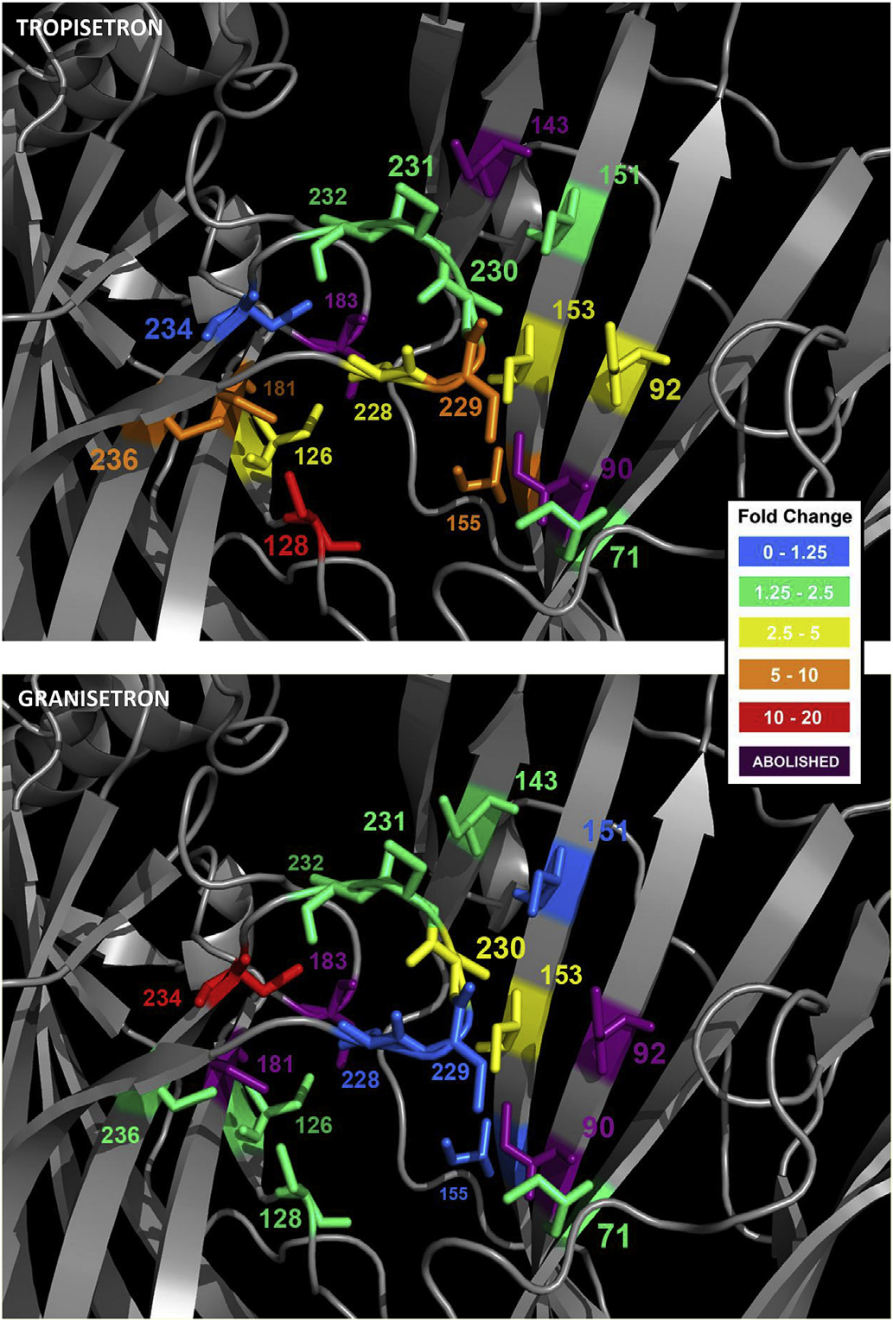
Binding site residues and the corresponding change in affinity when they are mutated. All positions in this figure are shown as cysteine and are colour coded according to the extent of the change in affinity this mutation caused. Residues that are thought to have a structural role have been omitted. The structure is *Model-1*, and more details of the effects of these cysteine substitutions can be found in Table 2, Table 3.

The binding affinity of [^3^H]tropisetron at each of the mutant receptors is shown in Table 2 and their locations in the 5-HT_3_ receptor binding site in Fig. 3. Changing 7 of the 23 residues caused no significant change in affinity when compared to wild type receptors, suggesting they do not have a significant role in ligand binding or the structure of the binding site (I71, Q151, T179, L184, S230, S231, N232). For the remaining 16 mutants there were differences in the binding affinities, indicating that these residues may interact with the ligand or alter the receptor structure. Of these, 9 had reduced affinities (R92, L126, N128, Y153, P155, T181, M228, E229, E236) and 7 showed no saturable binding (*K*_d_ > 20 nM; D69, W90, E129, Y143, W183, H185, Y234).

### 2.4 Effects of mutations on granisetron binding

Crystal structures of tropisetron or granisetron bound to the homologue AChBP (Fig. 2C) reveal very similar ligand orientations (Hibbs et al., 2009, Kesters et al., 2013). To determine whether these two ligands also adopt such similar poses in the 5-HT_3_ receptor we performed saturation binding with radiolabelled granisetron on the same mutants used for the tropisetron study. Of the 23 substitutions, 8 similarly affected granisetron binding, while the remaining 15 showed differing effects for the two ligands (Table 3, Fig. 3). Of those that were similar, the substitutions W90C, E129C and W183C abolished binding, Y153C reduced the affinity, and Q151C, L184C, S231C and N232C had no effect. In contrast to tropisetron, saturable binding of granisetron could be detected with D69C, Y143C and H185C, the mutants I71C, T179C and Y234C showed a significant decrease in affinity, S230C increased the affinity, R92C and T181C did not show saturable binding, and binding was unaltered by substitutions L126C, N128C, P155C, M228C, E229C, and E236C.

### 2.5 Western blot of non-binding mutants

**Fig. 4:**
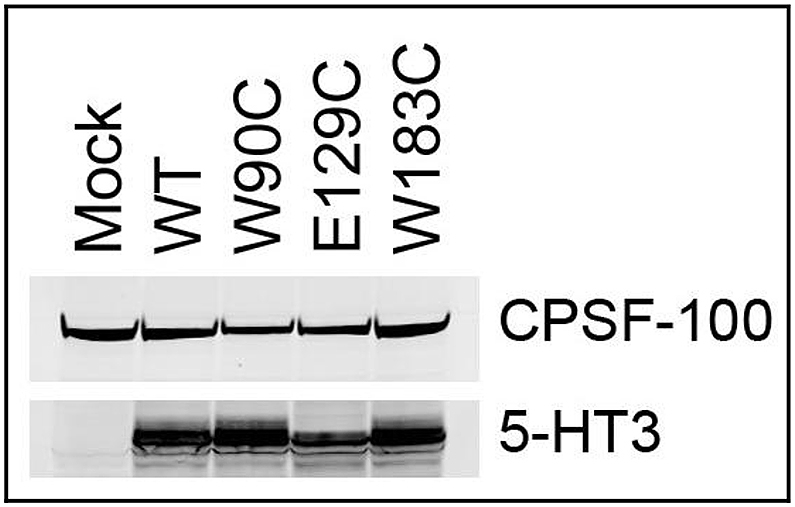
Expression of mutant 5-HT_3_ receptors. Three of the mutant receptors did not bind either [^3^H]tropisetron or [^3^H]granisetron (W90C, E129C, W183C). To confirm that this could be attributed to altered binding rather than changes in expression, these mutants were probed by Western blot using a 5-HT_3_ receptor-specific antibody. Expression of the mutants and wild type receptors were comparable, and there was no detection in untransfected cells. In each lane 10^5^ cell equivalents were loaded and an antibody against the cleavage and polyadenylation specificity factor (CPSF) included as an internal control. Note that whole-cell homogenates were used for both radioligand binding and Western blot to enable monitoring of the same populations of both intracellular and cell-surface receptors.

Three of the mutant receptors did not bind [^3^H]tropisetron or [^3^H]granisetron (W90C, E129C, W183C). To confirm that these receptors were expressed we performed Western blot with a 5-HT_3_ receptor-specific antibody. No immunostaining was apparent from untransfected HEK 293 cells, but clear bands were seen for wild type and mutant receptors at the expected size (Fig. 4).

### 2.6 Binding of granisetron & tropisetron derivatives

In addition to the changes made to the receptor, tropisetron and granisetron were also altered by synthesising derivatives (Table 4). It was clear from the affinities of these derivatives that modification of the bicyclic amine (*N*-8′) position was poorly tolerated by tropisetron, while substitution at the corresponding nitrogen of granisetron (*N*-9’) was less detrimental (see Table 4 for atom numbering). At the opposite end of the molecule, modifications at each of the three positions (C-5, C-6 and C-7) within the heteroaromatic ring of tropisetron reduced the binding affinity by 253-fold or less, while modification of only the C-5 position in granisetron caused a >10,000-fold decrease in affinity. These structure-activity relationships (SAR) of tropisetron and granisetron show that the two structurally similar ligands have different substitution tolerance patterns with respect to 5-HT_3_ receptor binding.

**Table 4:** Affinities determined from binding tropisetron, granisetron and their analogues.

Compound	R	R′	pK_i_ Mean ± SEM	K_i_ (nM)	n	Fold change
Tropisetron Derivatives						
Parent Scaffold	H	CH_3_	9.15 ± 0.06	0.72	8	–
1	5-OMe	CH_3_	7.02 ± 0.03	95.5	4	133
2	6-OMe	CH_3_	6.74 ± 0.04	182	4	253
3	7-OMe	CH_3_	7.52 ± 0.12	30.2	4	42
4	H	Bn	5.84 ± 0.20	1445	6	2007
Granisetron Derivatives						
Parent Scaffold	H	CH_3_	9.33 ± 0.11	0.47	6	–
5	5-OMe	CH_3_	5.27 ± 0.02	5370	4	>10,000
6	6-OMe	CH_3_	6.35 ± 0.18	447	5	951
7	7-OMe	CH_3_	6.92 ± 0.24	120	7	255
8	H	Bn	6.72 ± 0.24	190	6	404

* The structures below show tropisetron and granisetron with example saturations binding curves.

### 2.7 Binding at α7 nACh receptors

In contrast to its antagonist actions at 5-HT_3_ receptors, tropisetron is a partial agonist at α7 nACh receptors (Macor et al., 2001, Papke et al., 2005). A comparative study of its binding at these two receptor types could therefore provide insights into these distinct properties. However, saturation binding of [^3^H]tropisetron and [^3^H]granisetron could not be detected at α7 nACh receptors. In contrast, [^3^H]epibatidine bound with high affinity (*K*_d_ = 8.81 ± 0.13 nM, *n* = 5), allowing the affinities of non-radiolabelled tropisetron and granisetron to be probed by competition binding. For tropisetron this gave a *K*_i_ of 1.58 ± 0.84 μM (*n* = 3), while competition was not detected for granisetron at a concentration of up to 100 μM.

## 3 Discussion

Tropisetron and granisetron showed high-affinity, saturable binding at 5-HT_3_ receptors. For the majority (15/23) of cysteine substitutions we made to this receptor, the effects on binding were different for the two ligands, suggesting they may adopt different orientations. This observation was supported by our SAR on analogues of the same ligands, but conflicts with the very similar poses seen in co-crystal structures of these ligands bound to AChBP.

There have been several studies characterising the binding of orthosteric ligands at 5-HT_3_ receptors and a consensus regarding their function has been further guided by the more recent mouse 5-HT_3_ crystal structure 4PIR (Hassaine et al., 2014, Thompson et al., 2010). Some residues are thought to interact with the ligands, but others are thought to have primarily structural roles. From the crystal structure 4PIR it is clear that a hydrogen bond between E129 and T179 is structural (Hassaine et al., 2014). Such an interaction is supported by our finding that E129C abolishes binding of both ligands and by other reports that describe similar effects on granisetron, GR65630 and VUF10166 (Boess et al., 1997, Price et al., 2008, Thompson et al., 2005, Thompson et al., 2014). For T179C the effects on the two ligands were not as pronounced, but as both threonine and cysteine can act as hydrogen bond donors this property may be retained following substitution; consistent with this, substitution to serine also preserves granisetron binding (Thompson et al., 2005). L184 and H185 are also thought to make structural contributions by forming, respectively, hydrophobic interactions and a network of hydrogen bonds (Hassaine et al., 2014, Thompson et al., 2008). For L184 the absence of effects for both ligands were similar to previous reports of isoleucine substitution on granisetron binding, and as the sulphur in cysteine has a similar hydrophobic character to the side chains of leucine and isoleucine our result was anticipated (Nagano et al., 1999, Thompson et al., 2008). For H185 a substitution to cysteine would also conserve its side-chain interactions as both residues are capable of hydrogen bonding. However, it was previously reported that mutations at this location reduce cell-surface expression, and the lower B_max_ value we measured for granisetron (H185C = 123 ± 23 fmol mg^−1^; wild type = 4843 ± 512 fmol mg^−1^; paired samples, *n* = 4) is consistent with this (Joshi et al., 2006, Thompson et al., 2008). For tropisetron it is therefore possible that the absence of binding does not reveal interactions but, given the higher levels of non-specific binding, instead reveals expression levels that are below the threshold for detection.

**Fig. 5:**
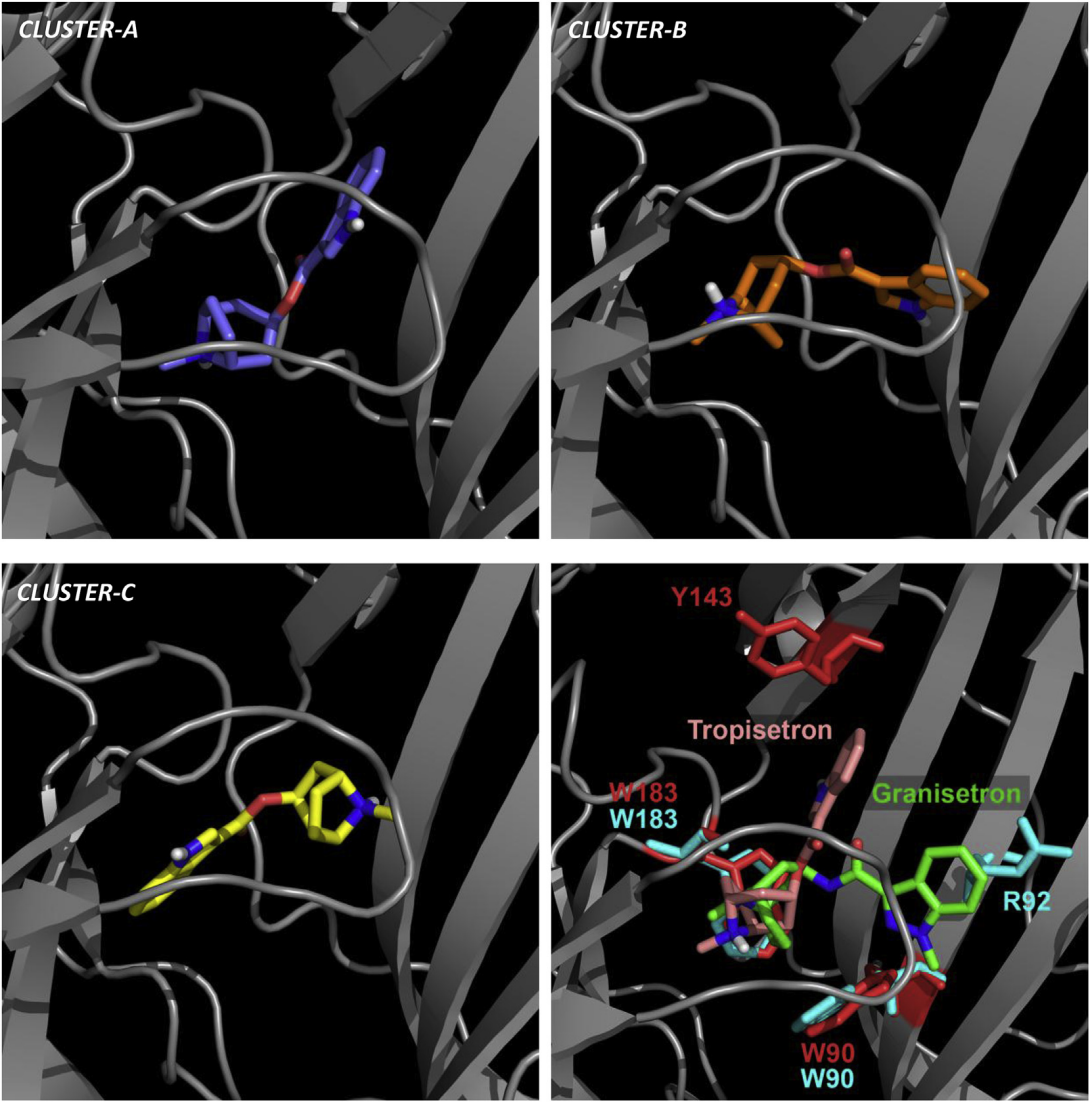
The three docked-pose clusters predicted by docking tropisetron into a homology model of the 5-HT_3_ receptor that was based on the template 2WNC (*Model-1*). Representative orientations of tropisetron are shown for each of the clusters *A*, *B* and *C* (for an overlay of all docked poses see Fig. 2A). In panel 5D, tropisetron (Clu*ster-A*, pink) , and granisetron (2YME, green) are overlaid. The residues shown are those that abolished binding in Fig. 3 (*Cluster-A*, red; 2YME, cyan). Other residues are omitted for clarity. For a full list of the identified residues in *Cluster-A* and 2YME see Table 1. The docked-pose predicted in *Cluster-A* is the most consistent with the binding results presented here. (For interpretation of the references to colour in this figure legend, the reader is referred to the web version of this article.)

In contrast to the structural roles described above, other binding site residues are thought to be responsible for direct ligand interactions. For some of these, the changes in affinity were large and provided compelling evidence of an interaction, particularly where the effects on tropisetron and granisetron differed. For example, the loop B mutant T181C caused a 6-fold decrease in affinity for tropisetron, but abolished granisetron binding. As 4PIR shows that this residue faces into the binding pocket, and mutations to smaller side-chains (Ala or Ser) are known to have no effect on granisetron binding, this difference may result from steric constraints that affect the two ligands differently (Thompson et al., 2008, Thompson et al., 2011, Thompson et al., 2014). In loop A, N128 also points towards the binding site, but consistent with the absence of specific interactions with the equivalent residue in 2YME (Y91), the affinity of granisetron was not altered. In previous work, a similar absence of effects on granisetron has also been reported for range of other substitutions at this location (Price et al., 2008, Sullivan et al., 2006). However, the effects of N128C we measured on the affinity of tropisetron (7-fold) and previous reports of changes to the *EC*_50_ of 5-HT, *m*CPBG and the binding affinity of VUF10166 suggests that effects may depend upon the ligand studied (Price et al., 2008, Sullivan et al., 2006, Thompson et al., 2014). In P4IR, N128 lies close to L126 on loop A, and E236 in loop C, none of which caused significant effects on granisetron binding. In contrast, tropisetron binding was altered by mutation at all of these locations (7-, 2.5- & 10-fold respectively, Table 2). When considered together this suggests that of the two ligands studied only tropisetron extends into this region of the binding pocket. Notably, neither L126 nor E236 are identified as lying within 5 Å of tropisetron in the tropisetron co-crystal structure 2WNC (Table 1). In contrast, both residues are predicted to lie within 5 Å of tropisetron in *Cluster-A*, suggesting that our mutagenesis is more consistent with this predicted orientation (Fig. 3, Fig. 5).

In loop D, R92C abolishes granisetron binding, but the affinity for tropisetron is reduced by only 2.5-fold. In the structure 2YME the equivalent residue (R55) makes a cation-π interaction with granisetron and this interaction is supported by both the effect we report here and by previous findings that showed preserving the charge in R92K mutants does not affect granisetron binding (Duffy et al., 2012, Kesters et al., 2013, Thompson et al., 2005). In sharp contrast, the much smaller change in affinity shown by tropisetron suggest that interactions at R92 are less important. In loop E, substitution of the aromatic residue Y143C abolished tropisetron binding, but did not affect the binding of granisetron. In the AChBP crystal structures 2YME and 2WNC, neither of the ligands extends towards this residue and an effect would not be anticipated (Fig. 2). For granisetron the absence of an effect is consistent with the crystallographic evidence, but for tropisetron it is not. Instead, our results are more consistent with the *in silico* predictions found in *Cluster-A* (Fig. 5), where tropisetron extends towards Y143, but is > 5 Å from R92. Indeed, the orientation of tropisetron in the crystal structure 2WNC might be considered atypical as other α7 nACh agonists such as nicotine (1UW6), varenicline (4AFT) and acetylcholine (3WIP) also display low affinity binding at 5-HT_3_ receptors and extend towards this region of the binding site. D69C also abolished tropisetron binding, but did not alter the binding affinity of granisetron. However, D69 lies outside of the recognised binding loops and, although it was identified as lying within 5 Å of tropisetron in a limited number of the docked poses, it does not have an obvious role in our 5-HT_3_ receptor homology models or the AChBP crystal structures. A more likely explanation is that, similar to H185C, the poor expression levels revealed by granisetron (D69C = 119 ± 16 fmol mg^−1^; wild type = 4843 ± 512 fmol mg^−1^; paired samples, *n* = 4) may be below the detection threshold for tropisetron.

Other mutations abolished the binding of both ligands. For example, the effect of the aromatic loop B residue W183C was anticipated for both tropisetron and granisetron as it is centrally located in the binding pocket and strongly influences the binding of several other 5-HT_3_ receptor ligands (Thompson et al., 2010). In loop C, Y234 also contributes to the aromatic nature of the binding site, and our finding that tropisetron binding was abolished and the affinity of granisetron was reduced (12-fold) is consistent with reports that aromatic properties at this position are important (Beene et al., 2004). In all of the models and the AChBP crystal structures presented here, the aromatic residue Y234, or its equivalent (Y193), interacts with tropisetron and granisetron (Kesters et al., 2013). As a similar side chain orientation is seen in 4PIR, ligand interactions with this residue are also likely in the 5-HT_3_ receptor binding site and are supported by the large effects we see for both ligands (Kesters et al., 2013). Substitution of another aromatic residue, W90 in loop D, also abolished the binding of both tropisetron and granisetron, similar to reports for other 5-HT_3_ receptor ligands (Thompson et al., 2014, Venkataraman et al., 2002, Yan et al., 1999). Again, the aromatic character of W90 is important as removal of the aromatic ring (W90A, W90S) eliminates granisetron binding, but conservative substitutions (W90Y) have reduced effects (Price and Lummis, 2004, Spier and Lummis, 2000, Thompson et al., 2005, Yan and White, 2005). In contrast to the residues described above, I71C lies outside of the recognised binding loops but is found within 5 Å of tropisetron in a limited number of the docked poses and 2WNC, and within 5 Å of granisetron in 2YME (Table 1). Nevertheless, there are no obvious ligand interactions predicted with this residue in our models or the crystal structures and the similar changes in affinity seen for both ligands (1.9 & 3.4-fold) may reveal a non-specific effect. P155 also lies outside of the recognised binding loops, but might be anticipated to impact binding as prolines often distort protein backbones. It is not clear from the structural evidence why the affinity of granisetron was unaffected and tropisetron showed a 5-fold decrease, but it is possible that by distorting the backbone the positions and side-chain orientations of the other interacting residues are affected.

When residues that significantly affected tropisetron binding were compared to the residues predicted to lie within 5 Å of this ligand, the docked poses in *Model-1* were the most consistent with the pattern of changes we observed. For example, residues L126 (loop A), T181, H185 (B), E236 (C) and Y143 (E) significantly altered the affinity for tropisetron and were also predicted in *Model-1*, while none of these were identified in either 2WNC or the poses predicted by docking tropisetron into 2YME (Table 1). Docked poses in *Model-1* fell into 3 main clusters, and *Cluster-A* was the largest of these (Fig. 5). This cluster contained the most residues that affected binding and was the only cluster to predict that tropisetron extends towards Y143, a loop E substitution that abolished tropisetron binding. This cluster also correctly excluded residues I71 and S231 which caused little or no change when substituted (Table 2, Fig. 3). Although 4 other residues (T179, L184, Q151, S230) were identified as being within the 5 Å docking sphere of *Cluster-A* but had no effects on binding, none of these were predicted to directly interact with tropisetron; T179 and L184 are likely to have a structural role (discussed above), the side chain of S230 is predicted to form a hydrogen-bond with the indole NH of tropisetron that would be retained with a cysteine substitution, and Q151 does not make specific interactions with the ligand. Furthermore, in *Cluster-A*, the bicyclic tropane moiety of tropisetron is located close to W183, consistent with the cation-π interaction that unnatural amino acid mutagenesis has identified for both granisetron and ondansetron at this location (Duffy et al., 2012). As related molecules that contain a similar moiety also inhibit the 5-HT_3_ receptor, such as tropine and atropine, retention of this interaction also seems quite likely for tropisetron (Lochner and Thompson, 2016, Papke et al., 2005). Our mutagenesis is therefore most consistent with the position of tropisetron predicted by *Cluster-A*; although we stress that its exact orientation may differ to that shown as our docking could be biased by the modelling template.

In contrast to tropisetron, the residues that affected granisetron binding were more limited in number and many were located at different positions (Fig. 3). Comparing the residues that affected binding with those predicted to interact with the ligand it is clear that the position of granisetron in 2YME is more consistent with the locations of the substitutions that altered its binding affinity. For example, of the 9 residues identified as being within 5 Å of granisetron in 2YME (Table 1), 6 of these (I71, W90, R92, Y153, W183, Y234) significantly reduced the affinity of granisetron (Table 3). Further, another 10 residues that are not within 5 Å of granisetron in 2YME also had no effect on binding (D69, L126, Y143, P155, L184, H185, M228, E229, N232, E236). For granisetron, we suggest that the position of the ligand in 2YME is broadly consistent with our 5-HT_3_ receptor mutagenesis.

By synthesising tropisetron and granisetron derivatives we were also able to probe binding by ligand SAR. Tropisetron was more tolerant to aromatic substitutions at the C-5, C-6, C-7 indole positons, while incorporation of a benzyl group to the *N*-8′ position at the opposite end of the ligand was less tolerated (Table 4). This SAR is most consistent with the predicted orientation for tropisetron found in *Cluster-A* as the benzyl substituent lies in a sterically unrestricted region (Fig. 5). Further, in the co-crystal structure 2W8G, Ulens et al. (2009) describes a comparable orientation for cmp-35, a ligand that similarly contains a tropane bicycle conjugated to a benzyl ring. For granisetron, our results are consistent with reports showing that conjugation of a methoxy group to the C-5 position of granisetron is not permitted, but increased tolerance is seen as we move towards the C-7 position and the indazole *N*-1 (Vernekar et al., 2010). In 2YME the C-7 and indazole *N*-1 nitrogen of granisetron lies within a sterically unrestricted location (Fig. 2, Fig. 5) and we have previously noted that attachment of bulky fluorophores via long linkers to each position can be accommodated (Jack et al., 2015, Simonin et al., 2012). These results contrast with those for tropisetron, and unlike tropisetron, are consistent with the orientation in the crystal structure 2YME. However, this orientation of granisetron is harder to reconcile with the finding that a benzyl group on the sterically restricted azabicyic ring of granisetron is better tolerated than elaborations of the relatively unrestricted C-5 position. We therefore speculate that *N*-9’-benzyl granisetron adopts a different orientation to the parent ligand; indeed, our *in silico* docking suggests that the addition of the benzyl ring to granisetron could force it to adopt an orientation similar to that of the benzyl derivative of tropisetron (cmp-35) described above (data not shown). In summary, these results show that tropisetron and granisetron have quite distinct SAR and further support our proposal that these two ligands are orientated differently in the 5-HT_3_ receptor binding site.

Originally we had sought to probe the different orientations of tropisetron at 5-HT_3_ and α7 nACh receptors and gain insights into its distinct actions at these two receptor types. However, saturation binding of tropisetron was undetectable at α7 nACh receptors, and a low binding affinity (1.58 μM) was only revealed following competition with [^3^H]epibatidine. This low affinity is in contrast to the findings of Macor et al. (2001) who reported a *K*_i_ of 6.9 nM at α7 nACh receptors, but is closer to the value that might be expected for a low potency agonist (e.g. *EC*_50_ = 1.3 μM, Macor et al., 2001; 0.6 μM, Papke et al., 2005), and to the affinity (0.5 μM) that was measured for tropisetron at AChBP and then used for co-crystallisation (Hibbs et al., 2009). Currently there are no mutation studies to support specific binding-site interactions between tropisetron and α7 nACh receptors. However, the amino acid sequences of AChBP and α7 nACh receptors share much stronger amino acid conservation with each other than with the 5-HT_3_ receptor and it might be expected that their binding affinities would also be closer (Fig. 1). In contrast, of the key 5-HT_3_ receptor residues identified for tropisetron in our study, N128 (Tyr in 2WNC and α7nACh), Y143 (Val in 2WNC; Leu in α7 nACh) and MESSN in loop C (CCPEP in 2WNC; CCKEP in α7 nACh) are quite different to their equivalents in 2WNC and the α7 nACh receptor, and may account for the much higher affinity of 5-HT_3_ receptors when compared to the other two proteins. Indeed, the amino acid conservation and similar affinities at AChBP and the α7nACh receptor suggest that in fact 2WNC may better represent the binding orientation of tropisetron at the α7 nACh. For granisetron, AChBP and the α7 nACh receptor are less similar as this ligand has a *K*_d_ of 1 μM at AChBP (Kesters et al., 2013), whilst we found no detectable binding at α7 nACh receptors with concentrations of up to 30 μM. Several of the residues in loops A, C and D are potential candidates for this difference as these they make contact with granisetron and differ between all three receptors. In particular, the residues of loop D (YIWYRQY in 5-HT_3_; NIWLQMY IN α7 nACh; VYYEQQR IN 2WNC) are highly varied, and in the 5-HT_3_ receptor this loop makes direct contact with granisetron (R92 forms a cation-π interaction) but not with tropisetron. However, it must be stressed that these speculations require experimental support and in future substituting AChBP and 5-HT_3_ residues into the α7 nACh receptor in an attempt to recover the higher affinities might provide clues as to which are important.

Our results confirm that both tropisetron and granisetron interact with residues in the 5-HT_3_ receptor orthosteric binding site, but mutagenesis and SAR with synthetic derivatives show that they are likely to adopt distinct poses when they are bound. These results contrast with the almost identical orientations seen in co-crystal structures of these ligands bound to the structurally-related protein AChBP. Ligand-based virtual screening has become a popular tool to discover novel active compounds to modulate therapeutically interesting protein targets (Reymond et al., 2011). These approaches are based on the assumption that structurally similar molecules should exhibit similar binding interactions with the target protein and hence display the same biological activities (Thompson et al., 2017). Our study suggests that even the very close structural similarity between tropisetron and granisetron does not necessarily lead to the same binding orientation. In addition, AChBP is sometimes used as a model for different members of the Cys-loop family, but our results suggest caution may be needed when interpreting ligand orientations in these different receptor homologues.

## 4 Materials and methods

### 4.1 Radioligands

[^3^H]Tropisetron was synthesized (Metis Laboratories Inc, NY, USA) from its desmethyl analogue (0.5 mg) in a tritiomethylation reaction by heating at 80 °C for 2 h with 50 mCi [^3^H]methyl iodide in tetrahydrofuran (THF) (0.4 ml). After evaporation of the THF the residue was subjected to reversed phase chromatography (Kromasil 100 C18, 7 μm, 250 × 10 mm, 2 mL min^−1^) using 31% ACN in 0.1% TFA as eluent. The eluent was stripped off and the residue was dissolved in 12 mL absolute ethanol. The [^3^H]tropisetron had a specific activity of 73.5 Ci/mmol and radiochemical purity of >99%. [^3^H]granisetron (63.5 Ci/mmol) and [^3^H]epibatidine (55.8 Ci/mmol) were purchased from Perkin Elmer (Waltham, MA) and were >97% chemical purity.

### 4.2 Synthesis of analogues

All reactions were performed under an inert argon atmosphere. Anhydrous THF and CH_2_Cl_2_ were obtained by filtration through a system of alumina columns under a positive pressure of argon. Solvents were evaporated under reduced pressure at approximately 45 °C using a Büchi Rotavapor or under high vacuum on a Schlenk line. Reagents and solvents were purchased from Sigma-Aldrich, Acros, Alfa Aesar, Fischer Scientific or Hänseler and used without further purification. Reactions were monitored by thin layer chromatography (TLC) using aluminum sheets pre-coated with silica gel (Macherey-Nagel ALUGRAM Xtra SII, G/UV254). Detection was achieved by excitation with a UV light source (*λ*_max_ 254 nm) or by staining with vanillin spray, with subsequent heating. Flash column chromatography was carried out using silica gel from Sigma-Aldrich (pore size 60 Å, 230–400 mesh particle size) as the stationary phase. ^1^H and ^13^C NMR spectra were recorded at 300 and 400 MHz, and 75 and 100 MHz respectively, on a Bruker Avance 300 and Avance II 400. Chemical shifts are reported in δ (ppm) and are referenced to the residual solvent peak. The order of citation in parentheses is (1) multiplicity: s (singlet), d (doublet), t (triplet), m (multiplet) etc., (2) coupling constants (*J*) in Hz, (3) number of equivalent nuclei (by integration), and (4) assignment. Mass spectra and high resolution mass spectra (HRMS) were recorded on a ThermoScientific LTQ Orbitrap XL spectrometer consisting of a linear ion trap (LTQ) featuring a HCD collision cell, coupled to the Orbitrap mass analyzer, equipped with a nanoelectrospray ion source (NSI). MS and HRMS spectra were determined by the Mass Spectrometry Group at the Department of Chemistry and Biochemistry, University of Bern (PD Dr. S. Schürch). The purity of the compounds was determined by UPLC-MS on a Dionex Ultimate 3000 using a reversed-phase column Dionex Acclain RSLC, 120C18, 3 × 50 mm, 2.2 μm, 120 Å pore size, flow 1.2 mL/min. Following gradient was used: 30 s at 100% A, then 100% A to 100% D over 5 min (A = 100% H_2_O with 0.1% TFA, D = H_2_O/MeCN 10:90 with 0.1% TFA). Purity was determined by total absorbance at 214 nm.

The synthesis of substituted granisetron derivatives has been described previously (Vernekar et al., 2010). The synthesis of substituted tropisetron derivatives was achieved by following this general procedure: the corresponding indole-3-carboxylic acid (1 equiv.) was suspended in CH_2_Cl_2_. An excess of thionyl chloride was added and refluxed for 2 h under an argon atmosphere. After completion, the volatiles were removed and the solid co-evaporated with CH_2_Cl_2_. The solid was dried and used in the next step without purification. In a separate flask the tropine (1-2 equiv.) was dissolved in THF under argon and cooled to 0 °C. *n*-Butyllithium solution (2.5 M in hexanes) was added and the resulting solution was stirred for 30 min at 0 °C. The acid chloride intermediate previously prepared was dissolved in THF and added to the pre-cooled solution. The resulting mixture was stirred at 0 °C for 1 h and at room temperature overnight. MeOH was added and the solvents were removed under reduced pressure. The residue was dissolved in CH_2_Cl_2_, washed with water and dried over Na_2_SO_4_. After filtration the organic phase was evaporated under reduced pressure. The crude was purified by column chromatography.

5-Methoxy-tropisetron ((1*R*,3*r*,5*S*)-8-methyl-8-azabicyclo[3.2.1]octan-3-yl 5-methoxy-1*H*-indole-3-carboxylate) was synthesized according to the general procedure, using 5-methoxy-1*H*-indole-3-carboxylic acid (50 mg, 0.26 mmol), thionyl chloride (0.5 mL, 6.9 mmol, 26 equiv.), tropine (73 mg, 0.52 mmol, 2 equiv.) and *n*-butyllithium solution (2 equiv.). After purification by column chromatography (silica gel, CH_2_Cl_2_/MeOH 8:2 + 0.5% aq NH_3_), 5-methoxy-tropisetron was obtained as a white solid (27 mg, 33% yield). ^1^H NMR (DMSO-*d*_6_) δ 1.76 (d, *J* = 14.9, 2H, aliphatic-CH), 1.98–2.18 (m, 6H, aliphatic-CH), 2.25 (s, 3H, N-Me), 3.14 (br s, 2H, aliphatic-CH), 3.78 (s, 3H, -OMe), 5.10 (t, *J* = 5.1, 1H, -COO-CH-(CH_2_)_2_), 6.84 (dd, *J* = 2.5, *J* = 8.8, 1H, C(6)-H), 7.39 (d, *J* = 8.8, 1H, C(7)-H), 7.51 (d, *J* = 2.5, 1H, C(4)-H), 7.93 (d, *J* = 3.1, 1H, C(2)-H), 11.77 (s, 1H, NH). ^1^H NMR (CDCl_3_) δ 1.88 (d, *J* = 14.9, 2H, aliphatic-CH), 1.99–2.17 (m, 4H, aliphatic-CH), 2.23–2.35 (m, 2H, aliphatic-CH), 2.31 (s, 3H, N-Me), 3.19 (br s, 2H, aliphatic-CH), 3.80 (s, 3H, -OMe), 5.23 (t, *J* = 5.2, 1H, -COO-CH-(CH_2_)_2_), 6.84 (dd, *J* = 2.5, *J* = 8.9, 1H, C(6)-H), 7.39 (d, *J* = 8.2, 1H, C(7)-H), 7.64 (d, *J* = 2.4, 1H, C(4)-H), 7.74 (s, 1H, C(2)-H), 9.60 (s, 1H, NH). ^13^C NMR (CDCl_3_) δ 25.9, 36.4, 40.1, 55.9, 60.2, 66.2, 102.4, 108.6, 112.6, 113.9, 126.9, 131.2, 131.3, 155.9, 164.7. HRMS (ESI+): exact mass calcd for C_18_H_23_N_2_O_3_ [M+H]^+^ 315.1703, found 315.1707, Δ: 1.2 ppm. UHPLC (*λ* = 214 nm) *t*_R_ = 2.16 min, 99% purity.

6-Methoxy-tropisetron ((1*R*,3*r*,5*S*)-8-methyl-8-azabicyclo[3.2.1]octan-3-yl 6-methoxy-1*H*-indole-3-carboxylate) was synthesized according to the general procedure, using 6-methoxy-1*H*-indole-3-carboxylic acid (35 mg, 0.183 mmol), thionyl chloride (0.3 mL, 4.14 mmol, 22 equiv.), tropine (52 mg, 0.366 mmol, 2 equiv.) and *n*-butyllithium solution (2.05 equiv.). After purification by column chromatography (silica gel, CH_2_Cl_2_/MeOH 8:2+ 0.5% aq NH_3_), 6-methoxy-tropisetron was obtained as a white solid (21 mg, 36% yield). ^1^H NMR (DMSO-*d*_6_) δ 1.78 (d, *J* = 14.9, 2H, aliphatic-CH), 2.04 (br s, 4H, aliphatic-CH), 2.08–2.19 (m, 2H, aliphatic-CH), 2.28 (s, 3H, N-Me), 3.19 (br s, 2H, aliphatic-CH), 3.78 (s, 3H, -OMe), 5.07 (t, *J* = 4.9, 1H, -COO-CH-(CH_2_)_2_), 6.83 (dd, *J* = 2.3, *J* = 8.7, 1H, C(5)-H), 6.98 (d, *J* = 2.2, 1H, C(7)-H), 7.86 (d, *J* = 2.8, 1H, C(2)-H), 7.88 (d, *J* = 8.6, 1H, C(4)-H), 11.67 (s, 1H, NH). ^1^H NMR (CDCl_3_) δ 1.91 (d, *J* = 14.8, 2H, aliphatic-CH), 2.05–2.15 (m, 4H, aliphatic-CH), 2.22–2.35 (m, 2H, aliphatic-CH), 2.34 (s, 3H, N-Me), 3.20 (br s, 2H, aliphatic-CH), 3.83 (s, 3H, -OMe), 5.26 (t, *J* = 5.3, 1H, -COO-CH-(CH_2_)_2_), 6.88 (d, *J* = 2.1, 1H, C(7)-H), 6.93 (dd, *J* = 2.3, *J* = 8.7, 1H, C(5)-H), 7.74 (s, 1H, C(2)-H), 8.09 (d, *J* = 8.7, 1H, C(4)-H), 9.19 (s, 1H, NH). ^13^C NMR (CDCl_3_) δ 26.0, 36.6, 40.3, 55.8, 60.0, 66.5, 95.2, 109.3, 111.8, 120.3, 122.0, 130.0, 137.2, 157.2, 164.7. HRMS (ESI+): exact mass calcd for C_18_H_23_N_2_O_3_ [M+H]^+^ 315.1703, found 315.1701, Δ: −0.6 ppm. UHPLC (*λ* = 214 nm) *t*_R_ = 2.19 min, 99% purity.

7-Methoxy-tropisetron ((1*R*,3*r*,5*S*)-8-methyl-8-azabicyclo[3.2.1]octan-3-yl 7-methoxy-1*H*-indole-3-carboxylate) was synthesized according to the general procedure, using 7-methoxy-1*H*-indole-3-carboxylic acid (100 mg, 0.523 mmol), thionyl chloride (0.85 mL, 11.7 mmol, 22 equiv.), tropine (81 mg, 0.575 mmol, 1.1 equiv.) and *n*-butyllithium solution (1.1 equiv.). After two purifications by column chromatography (first: Alox neutral, CHCl_3_/MeOH to 99:1; second: silica gel, CH_2_Cl_2_/MeOH 8:2+ 0.5% aq NH_3_), 7-methoxy-tropisetron was obtained as a white solid (19 mg, 12% yield). ^1^H NMR (DMSO-*d*_6_) δ 1.73 (d, *J* = 14.6, 2H, aliphatic-CH), 2.00 (br s, 4H, aliphatic-CH), 2.04–2.16 (m, 2H, aliphatic-CH), 2.21 (s, 3H, N-Me), 3.09 (br s, 2H, aliphatic-CH), 3.94 (s, 3H, -OMe), 5.07 (t, *J* = 5.1, 1H, -COO-CH-(CH_2_)_2_), 6.78 (d, *J* = 7.8, 1H, C(6)-H), 7.11 (t, *J* = 7.9, 1H, C(5)-H), 7.62 (d, *J* = 8.0, 1H, C(4)-H), 7.79 (d, *J* = 3.0, 1H, C(2)-H), 12.07 (s, 1H, NH). ^1^H NMR (CDCl_3_) δ 1.94 (d, *J* = 14.9, 2H, aliphatic-CH), 2.03–2.21 (m, 4H, aliphatic-CH), 2.26–2.42 (m, 2H, aliphatic-CH), 2.37 (s, 3H, N-Me), 3.24 (br s, 2H, aliphatic-CH), 3.96 (s, 3H, -OMe), 5.27 (t, *J* = 5.2, 1H, -COO-CH-(CH_2_)_2_), 6.71 (d, *J* = 7.7, 1H, C(6)-H), 7.19 (t, *J* = 8.0, 1H, C(5)-H), 7.78–7.83 (m, 2H, C(2)-H and C(4)-H), 9.32 (s, 1H, NH). ^13^C NMR (CDCl_3_) δ 25.9, 36.5, 40.2, 55.5, 60.2, 66.5, 103.1, 109.8, 113.9, 122.7, 126.9, 127.5, 130.2, 146.3, 164.6. HRMS (ESI+): exact mass calcd for C_18_H_23_N_2_O_3_ [M+H]^+^ 315.1703, found 315.1704, Δ: 0.3 ppm. UHPLC (*λ* = 214 nm) *t*_R_ = 2.27 min, 91% purity.

*N-*8′-Benzyl-tropisetron ((1*R*,3*r*,5*S*)-8-benzyl-8-azabicyclo[3.2.1]octan-3-yl 1*H*-indole-3-carboxylate) was synthesized according to the general procedure, using 1*H*-indole-3-carboxylic acid (37 mg, 0.23 mmol), thionyl chloride (0.3 mL, 4.14 mmol, 18 equiv.), *endo*-8-benzyl-8-azabicyclo[3.2.1]octan-3-ol (50 mg, 0.23 mmol, 1 equiv.) and *n*-butyllithium solution (1.09 equiv.). After purification by column chromatography (silica gel, CH_2_Cl_2_/MeOH 95:5), *N-*8′-Benzyl-tropisetron was obtained as a white solid (18 mg, 22% yield). ^1^H NMR (DMSO-*d*_6_) δ 1.77 (d, *J* = 15.0, 2H, aliphatic-CH), 2.04–2.20 (m, 6H, aliphatic-CH), 3.14 (br s, 2H, aliphatic-CH), 3.55 (s, 2H, N-CH_2_-Ph), 5.16 (t, *J* = 4.6, 1H, -COO-CH-(CH_2_)_2_), 7.14–7.54 (m, 8H, Ar-H), 7.96–8.09 (m, 2H, Ar-H), 11.89 (s, 1H, NH). ^1^H NMR (CDCl_3_) δ 1.86 (d, *J* = 15.0, 2H, aliphatic-CH), 2.00–2.18 (m, 4H, aliphatic-CH), 2.30 (d, *J* = 14.6, 2H, aliphatic-CH), 3.23 (br s, 2H, aliphatic-CH), 3.59 (s, 2H, N-CH_2_-Ph), 5.25 (t, *J* = 5.2, 1H, -COO-CH-(CH_2_)_2_), 7.13–7.31 (m, 5H, Ar-H), 7.32–7.44 (m, 3H, Ar-H), 7.78 (d, *J* = 2.9, 1H, C(2)-H), 8.10–8.22 (m, 1H, Ar-H), 8.93 (s, 1H, NH). ^13^C NMR (CDCl_3_) δ 26.2, 36.8, 56.6, 58.3, 67.1, 109.3, 111.8, 117.6, 121.4, 122.2, 123.4, 126.1, 126.5, 128.6, 129.1, 130.9, 136.3, 164.5. HRMS (ESI+): exact mass calcd for C_23_H_25_N_2_O_2_ [M+H]^+^ 361.1911, found 361.1911, Δ: 0.0 ppm. UHPLC (*λ* = 214 nm) *t*_R_ = 2.59 min, 99% purity.

### 4.3 Site-directed mutagenesis

Mutagenesis was performed using the QuikChange method (Agilent Technologies Inc., California, USA). Cysteine residues were substituted for amino acids throughout each of the binding loops A - E (Fig. 1). To facilitate comparisons with previous work, we have used the numbering of the equivalent residues in the mouse 5-HT3A subunit (Q6J1J7).

### 4.4 Cell culture and transfection

Human embryonic kidney (HEK) 293 cells were grown on 90 mm round tissue culture plates as monolayers in DMEM/F12 (Gibco, Life Technologies, CA, USA) supplemented with 10% fetal bovine serum (FBS; Sigma Aldrich) at 37 °C in a moist atmosphere containing 5% CO_2_.

Human 5-HT3A subunit cDNA (Accession: P46098, kindly provided by J. Peters, Dundee University, UK) was cloned into pcDNA3.1 for expression in HEK293 cells. The α7 nACh receptor was a chimaera of the chick α7 nACh extracellular domain fused to the 5-HT_3_ transmembrane/intracellular domains as described by Eiselé et al. (1993) and was similarly cloned into pcDNA3.1. Cells were transiently transfected with either of these cDNA constructs using polyethyleneimine (PEI: 25 kDa, linear, powder, Polysciences Inc., Eppelheim, Germany). 30 μl of PEI (1 mg ml^−1^), 5 μg cDNA and 1 ml DMEM were incubated for 10 min at room temperature, added drop wise to a 90 mm plate of 70–80% conﬂuent HEK293 cells, and incubated for 2–3 days before use.

### 4.5 Radioligand binding

Transfected HEK 293 cells were scraped into 0.6 ml of ice-cold 10 mM HEPES buffer (pH 7.4) and frozen. After thawing, they were washed with HEPES buffer, homogenised by trituration through a 25 gauge (0.5 mm) needle, and 50 μg of cell suspension incubated in 0.5 ml HEPES buffer containing different concentrations of radioligand. Non-specific binding was determined using 100 μM granisetron (with [^3^H]tropisetron), 100 μM tropisetron (with [^3^H]granisetron), or 30 μM -/-nicotine (with [^3^H]epibatidine). Equilibrium reactions were incubated on ice for at least 1 h and terminated by vacuum filtration onto Whatman GF/B filters wetted with 0.3% polyethyleneimine, followed by two rapid washes with 2.5 ml ice cold buffer. Radioactivity was measured by scintillation in Ultima Gold XR (Perkin Elmer, Waltham, MA) using a Tri-Carb 2100 TR (PerkinElmer) scintillation counter. Final counts were monitored to ensure that binding never exceeded 10% of the added concentrations of radioligands. For mutants that showed poor expression (D69C, H185C), B_max_ values were compared using paired samples of wild type and mutant receptor prepared under identical conditions on the same day.

Competition binding at α7 nACh receptors (10 point) was determined by incubating preparations of HEK 293 cells transfected with the α7 nACh receptor in 0.5 ml HEPES buffer containing 7 nM [^3^H]epibatidine and differing concentrations of the competing ligands granisetron (maximal concentration used = 30 μM) or tropisetron (max = 300 μM). Non-specific binding was determined with 30 μM (-)/-nicotine. Equilibrium reactions were incubated on ice for at least 1 h and bound radioligand measured using the same method as described above.

### 4.6 Data analysis

All data were analysed using GraphPad Prism v5. Individual saturation binding experiments were fitted to the equation below and the fitted values averaged to yield mean ± sem of *n* independent experiments:

$$y=B_{\max}\times \left[L\right]\frac{K_{d}}{K_{d}+\left[L\right]}$$

where *B*_max_ is maximum binding at equilibrium, *K*_d_ is the equilibrium dissociation constant and [*L*] is the free concentration of radioligand. Affinities were compared using 1-way ANOVA and a Dunnett's Post-Test.

### 4.7 Detection

48 h post transfection, HEK293 cells were harvested in 10 ml phosphate buffered saline (137 mM NaCl, 10 mM Na_2_HPO_4_, 2.7 mM KCl, 2 mM KH_2_PO_4_, pH 7.4), centrifuged 5 min at 220 g and the pellets stored at −80 °C. Total extracts were prepared by resuspension of 1 × 10^7^ cells in 1 ml lysis buffer (10 mM Tris-HCl pH 7.5, 10 mM NaCl, 2 mM EDTA, 5 mM MnSO_4_, 4 × Halt Protease Inhibitor (Thermo Fisher Scientific), 0.2 mg ml^−1^ RNase A (Sigma-Aldrich), 500 U ml^−1^ Cyanase (RiboSolutions, TX, USA), 0.1% Triton X-100) followed by 30 min on ice. Extracts were supplemented with NuPage LDS buffer (Thermo Fischer Scientific) and heated at 70 °C for 10 min. 10^5^ cell equivalents were loaded on a 4–12% Bis-Tris NuPage gel and blotted on nitrocellulose using the iBlot system (ThermoFisher Scientific). 5-HT_3_ receptors were detected with 1:250 mouse monoclonal antibody (sc-390168; Santa Cruz Biotechnology, TX, USA) and 1:10,000 goat anti-Mouse IRDye800CW (925-32210; LI-COR Biosciences, NE, USA). CPSF-100 served as a loading control and was detected using 1:10,000 rabbit polyclonal antibody (kindly provided by Georges Martin & Walter Keller, University of Basel) and 1:10,000 goat anti-Rabbit IRDye680LT (925-68021, LI-COR Biosciences). Signals were improved using SuperSignal Western Blot Enhancer (Thermo Fischer Scientific) according to the manufacturer's instructions.

### 4.8 Modelling and docking

Using ClustalW, the protein sequence of the human 5-HT3A subunit (accession: P46098) was aligned with the amino acid sequences extracted from ligand-bound AChBP crystal structures (pdbid; 2WNC, 2YME). Five pentameric homology models were generated using Modeller 9.13 with default parameters and the best model selected using Ramachandran plot analysis.

The three-dimensional and protonated structure of tropisetron was constructed using Chem3D Pro v14.0 (CambridgeSoft, Cambridge, UK) and was based on the crystal structure of *N*1-methylated tropisetron extracted from the Cambridge Structural Database (CSD access code BEGLEG). The generated ligand was subsequently energy-minimised using the implemented MM2 force field.

The binding site in the 5-HT_3_ receptor was defined as being within 10 Å of the centroid of the aromatic side-chain of W183, a residue that is centrally located in the binding site and known to be important for the binding of competitive ligands (Thompson et al., 2010). Potential ligand-receptor interactions were identified in each of the following four structures, A) a co-crystal structure of tropisetron and AChBP (2WNC), B) a co-crystal structure of granisetron and AChBP (2YME), C) tropisetron docked into a 5-HT_3_ receptor homology model based upon 2WNC, and D) tropisetron docked directly into 2YME. Tropisetron was docked using GOLD Suite v5.3 (The Cambridge Crystallographic Data Centre, Cambridge, UK) with the GoldScore function and default settings. During docking the carbonyl linker of tropisetron was defined as rigid as it is known that the planarity and rigidity of this bond is crucial for high-affinity binding (Hibert, 1994). For each of the two homology models, ten docking poses were generated. For 2WNC and 2YME the native poses were used. This analysis yielded 22 independent ligand poses. All residues within 5 Å of the ligand were identified in each of the ligand poses and these residues and their potential hydrogen bond interactions visualised using PyMol v1.3.

## Appendix A Supplementary data

Chemical compounds Chemical compounds
