# Supplementary material for: The binding orientations of structurally-related ligands can differ; A cautionary note
Source: Neuropharmacology. 2017 Jun;119:48–61. doi: 10.1016/j.neuropharm.2017.01.023 (PMC5464333; doi:10.1016/j.neuropharm.2017.01.023)
Supplement: Chemical compounds [file mmc1.docx]

**Chemical compounds studied in this article**

Tropisetron (PubChem CID: 656665)

Granisetron (PubChem CID: 5284566)

Chemical compounds studied in this article but not in PubChem:

**5-Methoxytropisetron (cmpd 1 in table 4)**

IUPAC systematic name:

(1*R*,3*r*,5*S*)-8-methyl-8-azabicyclo[3.2.1]octan-3-yl 5-methoxy-1*H*-indole-3-carboxylate

SMILES: CN1[C@H]2C[C@H](OC(C3=CNC4=CC=C(OC)C=C43)=O)C[C@@H]1CC2

**6-Methoxytropisetron (cmpd 2 in table 4)**

IUPAC systematic name:

(1*R*,3*r*,5*S*)-8-methyl-8-azabicyclo[3.2.1]octan-3-yl 6-methoxy-1*H*-indole-3-carboxylate

SMILES: CN1[C@H]2C[C@H](OC(C3=CNC4=CC(OC)=CC=C43)=O)C[C@@H]1CC2

**7-Methoxytropisetron (cmpd 3 in table 4)**

IUPAC systematic name:

(1*R*,3*r*,5*S*)-8-methyl-8-azabicyclo[3.2.1]octan-3-yl 7-methoxy-1*H*-indole-3-carboxylate

SMILES: CN1[C@H]2C[C@H](OC(C3=CNC4=C(OC)C=CC=C43)=O)C[C@@H]1CC2

***N*8’-Benzyltropisetron (cmpd 4 in table 4)**

IUPAC systematic name:

(1*R*,3*r*,5*S*)-8-benzyl-8-azabicyclo[3.2.1]octan-3-yl 1*H*-indole-3-carboxylate

SMILES: O=C(O[C@@H](C1)C[C@@H]2CC[C@H]1N2CC3=CC=CC=C3)C4=CNC5=CC=CC=C54

**5-Methoxygranisetron (cmpd 5 in table 4)**

IUPAC systematic name:

5-methoxy-1-methyl-*N*-((1*R*,3*r*,5*S*)-9-methyl-9-azabicyclo[3.3.1]nonan-3-yl)-1*H*-indazole-3-carboxamide

SMILES: CN1C2=CC=C(OC)C=C2C(C(N([C@@H]3C[C@@H]4CCC[C@H](C3)N4C)[H])=O)=N1

**6-Methoxygranisetron (cmpd 6 in table 4)**

IUPAC systematic name:

6-methoxy-1-methyl-*N*-((1*R*,3*r*,5*S*)-9-methyl-9-azabicyclo[3.3.1]nonan-3-yl)-1*H*-indazole-3-carboxamide

SMILES: CN1C2=CC(OC)=CC=C2C(C(N([C@@H]3C[C@@H]4CCC[C@H](C3)N4C)[H])=O)=N1

**7-Methoxygranisetron (cmpd 7 in table 4)**

IUPAC systematic name:

7-methoxy-1-methyl-*N*-((1R,3r,5S)-9-methyl-9-azabicyclo[3.3.1]nonan-3-yl)-1*H*-indazole-3-carboxamide

SMILES: CN1C2=C(OC)C=CC=C2C(C(N([C@@H]3C[C@@H]4CCC[C@H](C3)N4C)[H])=O)=N1

***N*9’-Benzylgranisetron (cmpd 8 in table 4)**

IUPAC systematic name:

*N*-((1*R*,3*r*,5*S*)-9-benzyl-9-azabicyclo[3.3.1]nonan-3-yl)-1-methyl-1*H*-indazole-3-carboxamide

SMILES: CN1C2=CC=CC=C2C(C(N([C@@H]3C[C@@H]4CCC[C@H](C3)N4CC5=CC=CC=C5)[H])=O)=N1
